# The Study on the Effect of Waterborne Epoxy Resin Content on the Performance of Styrene–Butadiene Rubber Modified Micro-Surface Mixture

**DOI:** 10.3390/polym17091175

**Published:** 2025-04-25

**Authors:** Lihua Zhao, Wenhe Li, Chunyu Zhang, Xinping Yu, Anhao Liu, Jianzhe Huang

**Affiliations:** 1 School of Transportation Engineering, Dalian Jiaotong University, Dalian 116028, China; 15048624665@163.com (W.L.); 17866535035@163.com (X.Y.); liuah16@163.com (A.L.); 18104351049@163.com (J.H.); 2 Yunnan Highway Science and Technology Research Institute, Kunming 650051, China; chunyuzhang@163.com

**Keywords:** micro-surfacing, modified emulsified asphalt, waterborne epoxy resin, styrene–butadiene latex, pavement performance

## Abstract

Conventional micro-surfacing materials often delaminate, crack, or peel. These defects shorten pavement life. High-performance polymer-modified mixtures are essential for rapid pavement maintenance. We added waterborne epoxy resin (WER) at different dosages to styrene–butadiene rubber (SBR) to create a composite-modified micro-surfacing mixture. A series of laboratory comparative tests were conducted to investigate the effect of WER content on the overall performance of the WER-SBR micro-surfacing mixture. In addition, the microstructure of the mixtures was observed to analyze the mechanism by which the composite-modified emulsified asphalt enhances material performance, and the optimal WER dosage was determined. The results showed that higher WER content improved abrasion and rutting resistance but gains plateaued above 6% WER. Below 9% WER, mixtures showed good water stability; at 3–6% WER, they also maintained skid and low-temperature crack resistance. Notably, when the WER content was approximately 6%, the WER-SBR micro-surfacing mixture showed significantly reduced abrasion damage after exposure to freeze–thaw cycles, moisture, and salt spray conditions. SEM images confirmed that 6% WER creates a uniform asphalt film over aggregates, boosting mixture performance. Therefore, we recommend 6% WER. This study has developed a WER-SBR composite-modified emulsified asphalt micro-surfacing product with excellent overall performance. It holds significant practical value for extending pavement service life and improving road service quality.

## 1. Introduction

As an effective preventive maintenance technique for asphalt pavements, micro-surfacing offers several advantages, including convenient construction at ambient temperature, early traffic opening, and low energy consumption. However, in practical engineering applications, issues such as poor adhesion between emulsified asphalt and aggregates often lead to severe surface defects, including looseness and aggregate loss [1,2,3]. These problems limit the duration for which micro-surfacing can maintain a satisfactory pavement condition. High-latitude coastal regions face high humidity, salt spray, and temperature swings. These conditions cause freeze–thaw cycles and salt erosion, which shorten pavement life [4,5,6]. The lack of high-performance pavement maintenance materials has become a key constraint on the effectiveness of asphalt pavement preservation and urgently needs to be addressed. We need durable, easy-to-apply maintenance materials. Such materials cut life-cycle costs, boost vehicle efficiency, and reduce carbon emissions. As a result, this has become a major research focus in the field [7].

In micro-surfacing structures, the primary strength is derived from the bonding force between asphalt and aggregates rather than the interlocking effect among aggregates [8]. Therefore, polymer-modified emulsified asphalt is commonly used to prepare micro-surfacing mixtures [9,10], with the aim of improving material performance and extending service life. Common modifiers include styrene–butadiene rubber (SBR) [11], polyurethane (PU) [12], and styrene–butadiene–styrene block copolymer (SBS) [13]. SBR latex maintains good deformability under low-temperature conditions and exhibits favorable compatibility with other materials, leading to its widespread application [14]. Qu et al. incorporated powdered SBR into Buton rock asphalt and significantly improved the mixture’s low-temperature crack resistance and aging resistance. However, the addition of SBR negatively affected the mixture’s rutting resistance [15]. Han et al. found that the dosage of SBR had a substantial impact on the bonding properties of asphalt and the adhesion of the mixture. When the SBR content was 3%, the modified emulsified asphalt exhibited optimal low-temperature crack resistance [16]. Hu et al. investigated the effect of SBR latex content on the high- and low-temperature performance of emulsified asphalt and found that increasing the SBR latex content from 4% to 6% significantly increased the phase angle variation, indicating a decline in thermal stability due to excessive SBR addition [17]. These findings suggest that SBR-modified emulsified asphalt exhibits good low-temperature performance and ductility, making it suitable for cold, high-latitude regions. However, its high-temperature performance in micro-surfacing mixtures is notably inadequate [18,19]. Therefore, it is necessary to identify a new modifier to compensate for these shortcomings and improve the overall performance of micro-surfacing mixtures.

Waterborne epoxy resin (WER) emulsified asphalt has emerged in recent years as a promising new road material. It is formulated by blending WER, a curing agent, and emulsified asphalt in appropriate proportions [20,21]. This material offers several advantages, including environmental friendliness, ease of storage and transportation, and good workability. Upon mixing with the curing agent, WER forms a high-strength, three-dimensional cross-linked network, significantly enhancing the high-temperature performance of emulsified asphalt [22,23,24,25]. As a result, WER emulsified asphalt has been widely applied in bonding layers, pavement pothole repair, and surface wear layers. Numerous researchers have conducted extensive studies on its application characteristics [26,27,28]. Song, Wu, and others developed a novel method for preparing WER-modified emulsified asphalt and applied it to pavement bonding layers. Through a combination of microscopic characterization and macroscopic performance testing, they confirmed the thermosetting characteristics of epoxy resin [29,30]. Xu, Yang, and colleagues applied WER to pothole repair materials. Their experimental results demonstrated the feasibility of using WER-modified asphalt as a cold patching material. The study found that increasing the WER content improved the high-temperature and water stability performance of the cold patching mixture, though it negatively impacted low-temperature performance [31,32]. Liu, Han, and others used WER-modified emulsified asphalt in surface wear layers and evaluated its pavement performance and interlayer shear strength. Their findings indicated that WER effectively mitigates rutting and wear on pavements, significantly extending service life [33,34,35].

From the perspective of microscopic mechanisms, Kong et al. used microstructural tests to show that WER creates a honeycomb cross-linked network in asphalt, forming an epoxy-asphalt biphasic structure. They identified 20% as the optimal concentration of WER [36]. Sun et al. [37] investigated the curing process of low-content thermosetting epoxy asphalt (LTEA) using Fourier transform infrared spectroscopy (FTIR) and Marshall tests and explained the mechanism of strength formation. The study revealed that using LTEA as a binder reduced both material cost and carbon emissions by 24.8% and 26.3%, respectively, compared with traditional materials. Meng, Yao, and colleagues explored the modification mechanism of emulsified asphalt with WER and SBR. Through fluorescence microscopy and FTIR tests, they confirmed that SBR, WER, and emulsified asphalt together formed a well-developed spatial network structure. This structure significantly improved the heat resistance of the asphalt while maintaining good low-temperature flexibility [38,39].

WER-modified micro-surfacing offers high-temperature strength, abrasion, and skid resistance. SBR-modified mixtures provide low-temperature ductility. WER and SBR are compatible with emulsified asphalt and can be used in combination. At present, research on micro-surfacing mixtures using SBR and WER composite-modified emulsified asphalt remains at a preliminary stage. There is a limited scientific understanding of the effects of WER on the performance of SBR-based micro-surfacing mixtures. Challenges include the lack of precise control over the optimal dosages of the two modifiers and insufficient investigation into the mixtures’ performance under varying service conditions. This study focused on the overall performance of micro-surfacing mixtures and investigated the effect of different WER contents on SBR-modified mixtures. A series of laboratory tests were conducted to evaluate the wear resistance, rutting resistance, moisture stability, skid resistance, and low-temperature cracking resistance of the WER-SBR composite mixtures. Considering the conditions of high-latitude coastal regions-seasonal freezing and salt spray exposure-weathering tests under freeze–thaw, wet–dry, and salt spray environments were also designed to comprehensively assess the pavement performance of the mixtures. SEM tests were used to observe the microstructure of the WER-SBR micro-surfacing mixture and to reveal the reinforcement mechanism of the composite modifier. Based on both macro and micro-scale analyses, the optimal WER content in the SBR-modified system was determined. This study aims to develop a WER–SBR composite-modified emulsified asphalt micro-surfacing mixture with superior comprehensive performance. The results hold significant engineering importance and theoretical value for extending the service life of micro-surfacing mixtures and enhancing the effectiveness of highway maintenance.

## 2. Materials and Methods

### 2.1. Materials

#### 2.1.1. Emulsified Asphalt

In this study, BC-1 type slow-breaking cationic base emulsified asphalt was selected. It is black-brown in color and is primarily used in pavement micro-surfacing maintenance. The technical specifications are shown in Table 1 and meet the requirements of the relevant technical standards [40].

#### 2.1.2. Modifier

In this study, two types of modifiers, SBR latex and the WER system, were used to modify the base emulsified asphalt. The SBR latex is cationic in nature, sourced from Europe, and is milky white in appearance with a measured pH value of 3.5. It shows good compatibility with BC-1 type cationic emulsified asphalt. The technical specifications of the WER system are shown in Table 2, with a ratio of WER to curing agent of 2:1.

#### 2.1.3. Aggregates

In this study, both coarse and fine aggregates were made of limestone. Their performance indicators, as shown in Table 3, meet the requirements of the relevant technical standards [40].

#### 2.1.4. Fillert

In this study, ground limestone powder and P·O42.5 ordinary Portland cement were used as fillers. Based on multiple mixing trials, the optimal dosages were determined as follows: 4% limestone powder, 2% cement, and 7% mixing water. This combination ensured good workability and sufficient strength of the micro-surfacing mixture. The technical properties of the limestone powder and cement are shown in Table 4, and all meet the relevant standards [41,42].

### 2.2. Gradation

**Figure 1:**
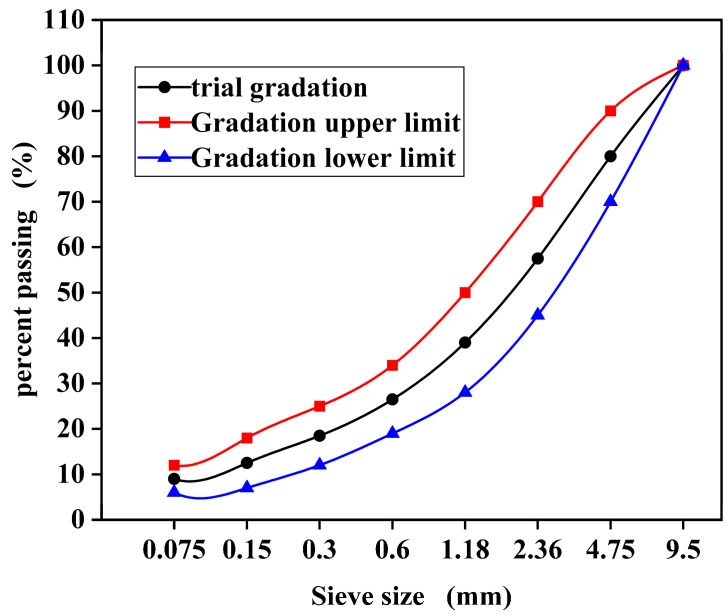
Gradation curve of micro-surfacing.

The aggregate gradation of micro-surfacing has a significant impact on the overall performance of the mixture [43]. Currently, the commonly used gradation types for micro-surfacing mixtures in China are MS-II and MS-III. Based on preliminary mix design trials, the median gradation of the MS-III type was selected for this study. The gradation curve is shown in Figure 1.

### 2.3. Experimental Design

**Figure 2:**
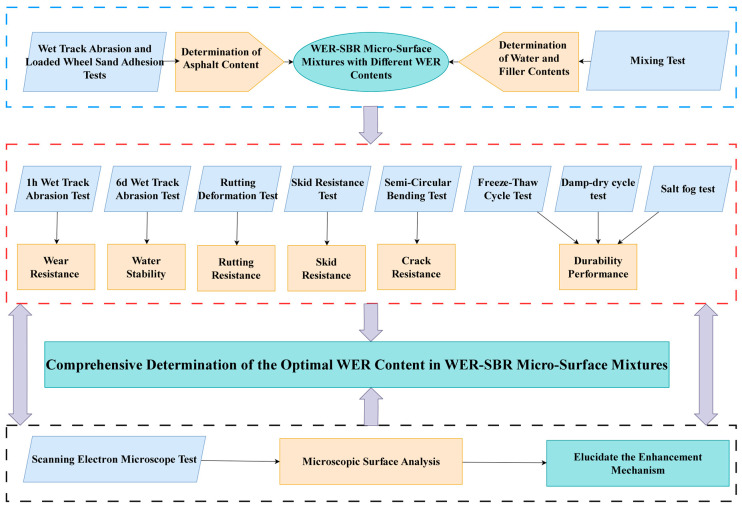
Technical route diagram.

This study investigates the influence of WER content on the overall performance of SBR-based micro-surfacing mixtures, with the aim of developing a product featuring excellent high- and low-temperature performance, skid resistance, and wear resistance, suitable for application in seasonally frozen coastal regions at high latitudes. The experimental technical route is illustrated in Figure 2.

In this study, composite-modified emulsified asphalt was prepared using the external addition method. Previous research by our team [44] and other studies [14,45] have shown that a 3% SBR content offers a good balance between low-temperature performance and cost-effectiveness. The WER system exhibits favorable high-temperature and bonding properties at lower dosages [46,47]. Therefore, WER was added to SBR-modified emulsified asphalt at contents of 3%, 6%, 9%, and 12% to prepare WER-SBR composite micro-surfacing mixtures. Their overall performance was then evaluated. Based on 100 g of aggregate as the reference, the material proportions for each experimental group are listed in Table 5.

**Figure 3:**
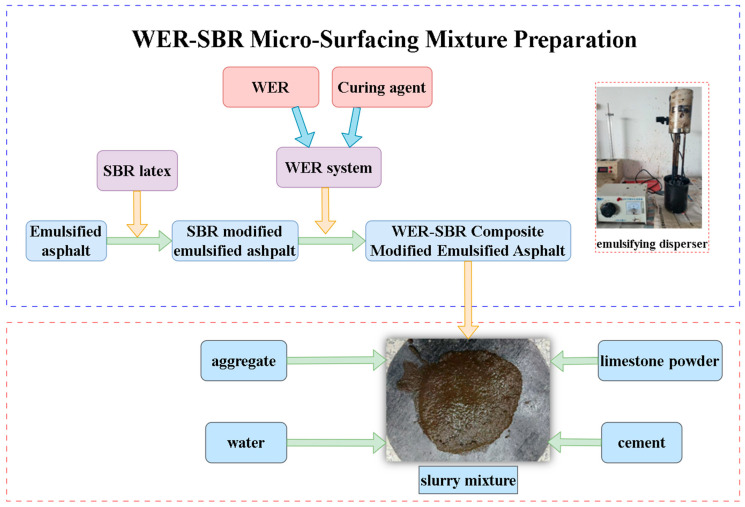
Flowchart of WER-SBR micro-surfacing mixture preparation.

The preparation process of the WER-SBR micro-surfacing mixture is as follows:

A small amount of BC-1 base emulsified asphalt is measured and mixed with 3% SBR latex by weight. The mixture is stirred using a high-shear emulsifier (as shown in Figure 3) at a shear rate of 500 r/min for 2 min to produce SBR-modified emulsified asphalt.

WER and curing agent were mixed at a 2:1 ratio and stirred at 500 r/min for 2 min using a shear mixer to form the waterborne epoxy system. This system was then added to the SBR-modified emulsified asphalt at different dosages and sheared again at 500 r/min for 2 min to produce the WER-SBR composite-modified emulsified asphalt.

The aggregates were sieved according to the target gradation and mixed with cement and mineral powder. After that, the predetermined amount of water was added and stirred. Finally, the calculated amount of WER-SBR composite-modified emulsified asphalt was added and mixed for 30 s to produce the slurry mixture.

The preparation process is illustrated in Figure 3.

### 2.4. Determination of the Asphalt–Aggregate Ratio of Micro-Surfacing Mixture

According to the technical specifications [40], the asphalt content range for micro-surfacing mixtures is determined based on both the Wet Track Abrasion Test (WTAT) and the Loaded Wheel Sand Adhesion Test (LWT). Mixture specimens were prepared using six asphalt–aggregate ratios: 6.5%, 7.0%, 7.5%, 8.0%, 8.5%, and 9.0%. The material quantities for each group of specimens are listed in Table 6. The testing procedures were conducted in accordance with the relevant standards [48]. The WTAT value was calculated using Equation (1), and the LWT value was calculated using Equation (2).

**Table 6:** Material quantities for each test group with different asphalt–aggregate ratios.

Asphalt–Aggregate Ratio (%)	Dry Material ^1^ (g)	Water (g)	BC-1 Emulsified Asphalt (g)
6.5	106	7	6.5
7	106	7	7
7.5	106	7	7.5
8	106	7	8
8.5	106	7	8.5
9	106	7	9

^1^ Dry material includes aggregate, mineral filler, and cement.

(1) $$WTAT=\frac{m_{b}-m_{a}}{A}$$

WTAT = wet wheel abrasion value (g/m^2^); m_a_ = mass of the specimen before abrasion (g); m_b_ = mass of the specimen after abrasion (g); A = abrasion area (m^2^).

(2) $$LWT=\frac{m_{2}-m_{1}}{A}$$

LWT = Sand adhesion amount (g/m^2^); m_2_ = total mass of the sand and specimen after 100 rolls (g); m_1_ = mass of the specimen after 1000 rolls (g); A = rolling area (m^2^).

### 2.5. Wet Wheel Abrasion Test

**Figure 4:**
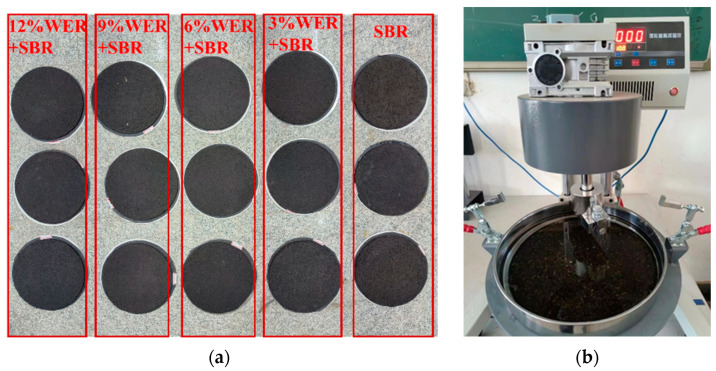
Specimen of wet wheel abrasion and wet wheel abrasion tester: (**a**) specimen; (**b**) instrument.

In this study, the abrasion resistance and moisture stability of the WER-SBR micro-surfacing mixture were evaluated using the Wet Track Abrasion Test (WTAT) after water immersion for 1 h and 6 days. The WTAT parameter was used for performance assessment. Specimens with varying dosages of the WER system were prepared, as shown in Figure 4a. Each specimen had a diameter of 280 mm and a thickness of 6.5 mm. The abrasion test was conducted using the setup illustrated in Figure 4b, following the procedures specified in the standard [48]. The WTAT value was calculated using Equation (1).

### 2.6. Rutting Deformation Test

**Figure 5:**
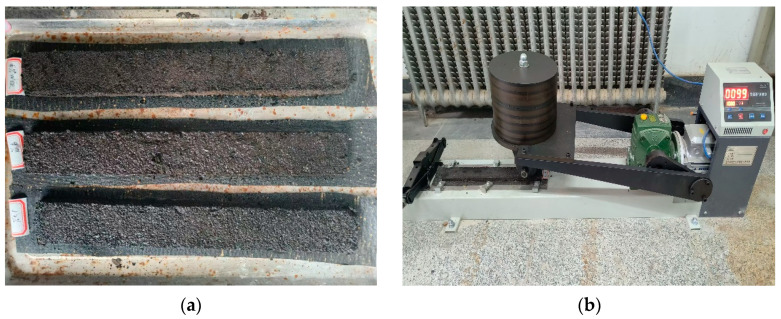
Specimen of load wheel tests and load wheel testing apparatus: (**a**) specimen, (**b**) instrument.

The rutting resistance of the WER-SBR micro-surfacing mixture was investigated through the rutting deformation test. The evaluation was based on the Percent Longitudinal Deformation (PLD) and Percent Vertical Deformation (PVD). Specimens with different dosages of the WER system were prepared for testing, as shown in Figure 5a. The initial width (L_a_) and thickness (D_a_) of each specimen were measured. The specimens were then placed in the loading wheel testing apparatus, as illustrated in Figure 5b, and subjected to 1000 passes of wheel loading. After the test, the final width (L_b_) and thickness (D_b_) were recorded. The values of PLD and PVD were calculated using Equations (3) and (4), respectively.

(3) $$PLD=\frac{\left(L_{b}-L_{a}\right)\times 100}{L_{a}}$$

(4) $$PVD=\frac{D_{b}\times 100}{D_{a}}$$

where PLD = width deformation rate (%); PVD = depth deformation rate (%) of the micro-surfacing rutting specimen.

### 2.7. Anti-Skid Performance Test

**Figure 6:**
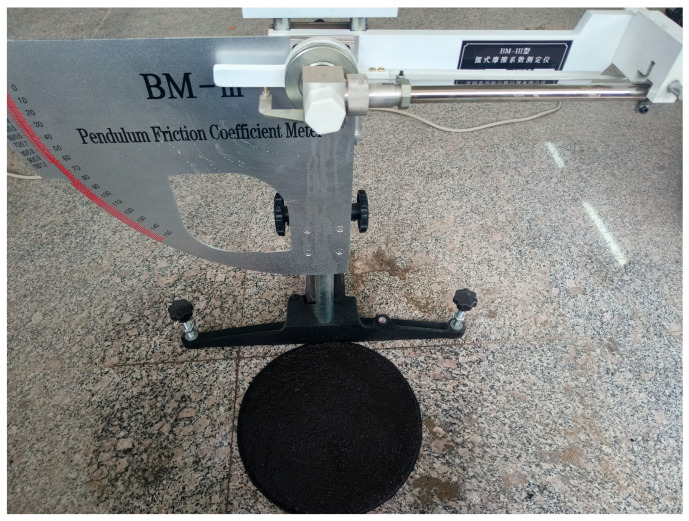
BM-III type pendulum friction tester.

The skid resistance of the WER-SBR micro-surfacing mixture was evaluated using the British Pendulum Number (BPN). The test was conducted with a BM-III pendulum friction tester (Dalian Jiaotong University, Dalian, China), as shown in Figure 6. Wet track abrasion specimens were prepared and cured for 48 h. Prior to testing, the specimen surface was cleaned and wetted with water. The specimen was then positioned directly beneath the pendulum device, and the test was initiated. The BPN value was recorded at the point where the pendulum ceased movement after sliding across the specimen surface. BPN values were measured for mixtures prepared with different dosages of the WER system. In addition, BPN was recorded after 30, 60, 90, and 120 min of wet track abrasion to assess the change in skid resistance over time. These results were compared with those of mixtures modified only with SBR latex to evaluate the effects of WER dosage and abrasion duration on the skid resistance of the micro-surfacing mixture.

### 2.8. Semi-Circular Bending Test

**Figure 7:**
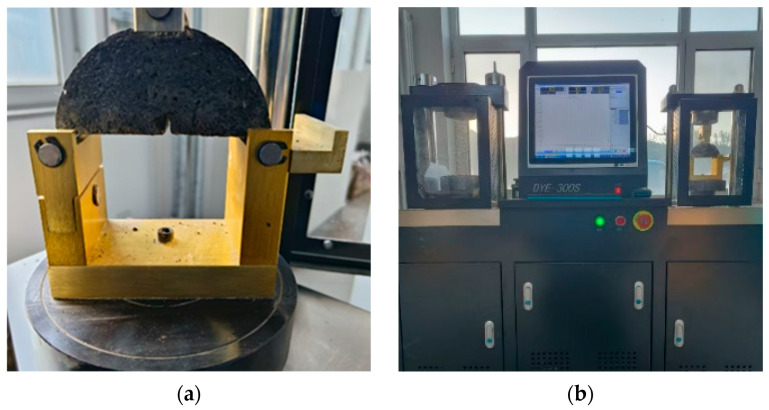
Load wheel testing apparatus: (**a**) semi-circular bending specimen; (**b**) DYE-300S Series Electronic Universal Testing Machine.

The low-temperature crack resistance of the WER-SBR micro-surfacing mixture was studied using the semi-circular bending (SCB) test, and the bending strength was used as the evaluation parameter. First, Marshall specimens of the WER-SBR micro-surfacing mixture were prepared. The specimens were then cut radially using a high-precision cutting machine to obtain semi-circular bending specimens with a pre-fabricated notch at the bottom of the specimen, as shown in Figure 7a. The specimens were subsequently loaded using a DYE-300S series testing machine (Dalian Jiaotong University, Dalian, China), as shown in Figure 7b. The maximum load during the fracture process was recorded. The bending strength of the specimens was calculated using Equation (5).

(5) $$\sigma =\frac{3PL}{2{\mathrm{th}}^{2}}=\frac{6PL}{tD^{2}}$$

where P = load (N); L = span between supports (mm); h = specimen height (D/2) (mm); t = specimen thickness, typically taken as 40 mm; σ = bottom tensile stress value of the specimen (MPa).

### 2.9. Freeze–Thaw Cycle Test

**Figure 8:**
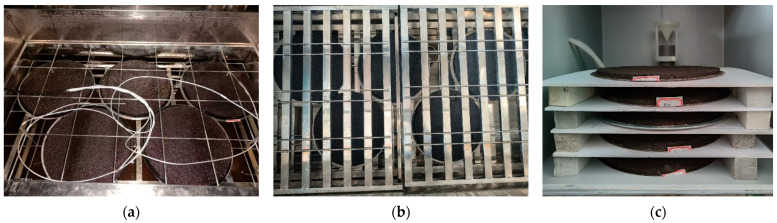
Wet track abrasion specimens in different test chambers: (**a**) freeze–thaw cycle chamber; (**b**) dry–wet cycle chamber; (**c**) salt spray test chamber.

This study investigated the freeze–thaw resistance of the WER-SBR micro-surfacing mixture through freeze–thaw cycle testing, using the Wet Track Abrasion Test (WTAT) after the freeze–thaw cycles to evaluate its performance in cold environments. First, wet track abrasion specimens with different dosages of WER were placed in the freeze–thaw cycle chamber, as shown in Figure 8a. The temperature cycle conditions were set to range from −20 °C to 25 °C, with a curing period of 3 days. After curing, the specimens were subjected to the Wet Track Abrasion Test, and the WTAT values were calculated using Equation (1).

### 2.10. Damp–Dry Cycle Test

This study investigated the service performance of the WER-SBR micro-surfacing mixture in a hot-humid environment through dry–wet cycle testing, using the Wet Track Abrasion Test (WTAT) after the dry–wet cycles for evaluation. Wet track abrasion specimens with different dosages of WER were prepared and placed in the dry–wet cycle test chamber, as shown in Figure 8b. The temperature cycle conditions were set to range from 30 °C to 80 °C, with cycle durations of 24 h, 48 h, and 72 h. After completing the cycles, the specimens were subjected to the Wet Track Abrasion Test, and the WTAT values were calculated using Equation (1).

### 2.11. Salt Fog Test

In response to the seasonal freezing climate and salt fog environment in coastal areas, the salt fog resistance of the micro-surfacing mixture was evaluated using the Wet Track Abrasion Test (WTAT) after salt fog exposure. In this study, a salt fog test chamber with adjustable temperature (TMS9024) was used to condition the specimens. Wet track abrasion specimens with different dosages of the WER system were placed inside the salt fog chamber, as shown in Figure 8c. A 5% NaCl solution was added to the water tank to generate the salt fog. The test was conducted at temperatures of 15 °C, 25 °C, and 35 °C with a salt fog humidity of 90%. After 2 days of exposure, the specimens were removed and subjected to the Wet Track Abrasion Test, with the WTAT values calculated using Equation (1).

### 2.12. Scanning Electron Microscope (SEM) Test

**Figure 9:**
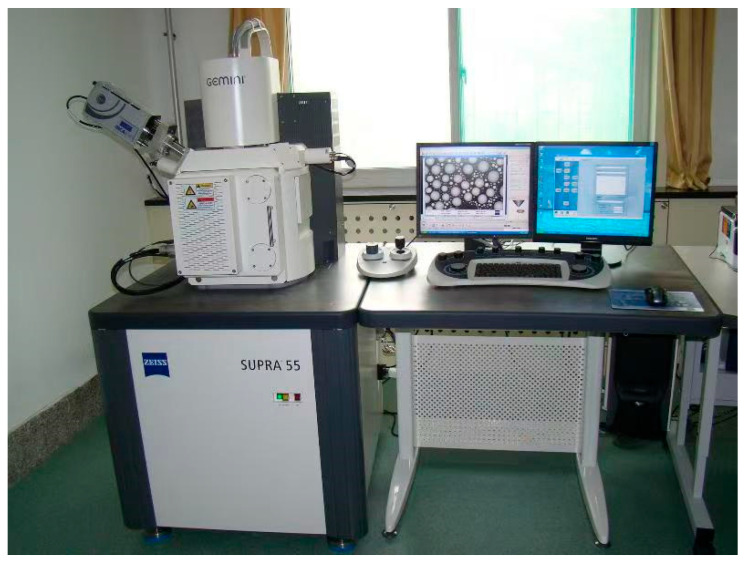
SEM testing instrument.

The performance of micro-surfacing mixtures is influenced by multiple factors, including aggregate gradation, overlay thickness, temperature, and humidity. Therefore, predicting the actual in-service behavior solely based on the microscopic mechanisms of the asphalt phase is not sufficient. Hence, it is essential to conduct a microscopic analysis from the perspective of the mixture. In this study, scanning electron microscopy (SEM) was used to observe the microstructure of the WER-SBR micro-surfacing mixture and to reveal the improvement mechanism of the WER system on the mixture’s performance. The SEM specimens were taken from newly formed wet track abrasion samples and observed using a German Zeiss SUPRA 55 field emission scanning electron microscope (Dalian Jiaotong University, Dalian, China), as shown in Figure 9. Prior to testing, the specimens were gold-coated. The observations were made at a magnification of 200 times, with a test voltage of 5 kV.

## 3. Results and Discussion

### 3.1. Results of the Asphalt–Aggregate Ratio of the Micro-Surfacing Mixture

**Figure 10:**
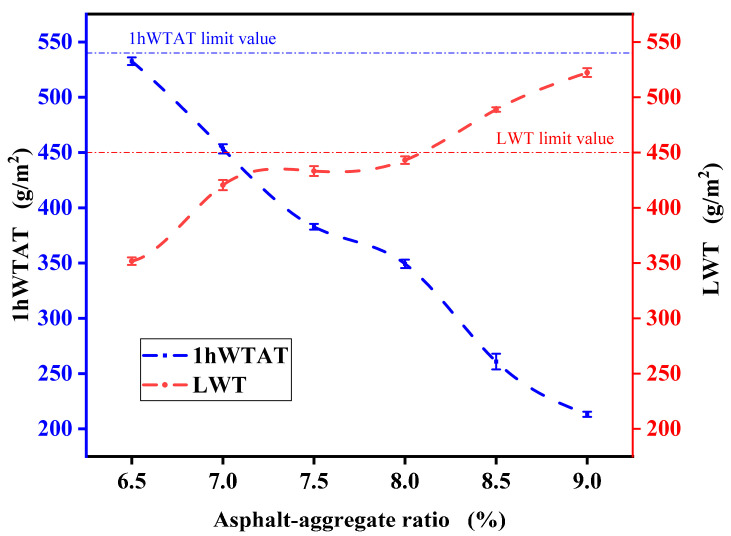
Asphalt–aggregate ratio range curve.

Figure 10 shows the WTAT and LWT of the micro-surfacing mixture at different asphalt–aggregate ratios.

**Figure 11:**
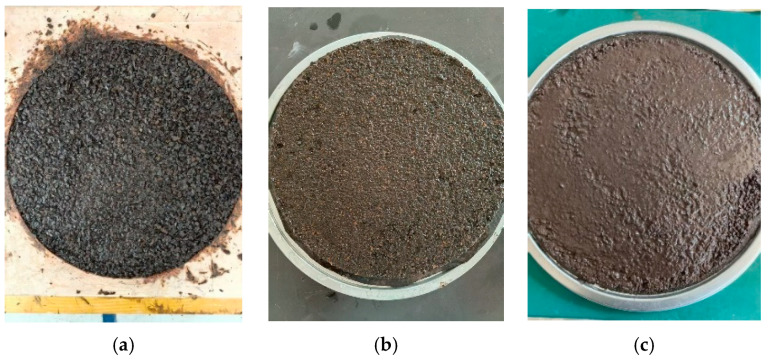
Specimen surfaces with different asphalt–aggregate ratios: (**a**) 6.5%; (**b**) 7%; (**c**) 9%.

The experimental results indicate that with the increase in the asphalt-to-aggregate ratio, the 1 h WTAT value of the mixture gradually decreases while the LWT value increases. According to the specifications, the 1 h WTAT value for micro-surfacing mixtures should be less than or equal to 540. Within the tested range, all WTAT values complied with this requirement. As the WTAT value approaches 540, a significant amount of aggregate remains uncoated by asphalt, as shown in Figure 11a, indicating that the asphalt content has reached its critical minimum. With further increases in asphalt content, more aggregates become coated and contribute to the formation of mixture strength, resulting in a denser structure, as illustrated in Figure 11b. Additionally, the enhanced bonding performance of the composite-modified emulsified asphalt improves the mixture’s cohesion, leading to a gradual increase in the LWT value. The specification requires that the LWT value not exceed 450. When the LWT approaches this limit, a higher proportion of slurry appears in the mixture, and asphalt bleeding occurs on the specimen surface, as shown in Figure 11c, indicating the critical maximum asphalt content.

As shown in Figure 10, when the asphalt-to-aggregate ratio exceeds 6.5%, the 1 h WTAT value meets the standard, and when it is below 8%, the LWT value is within the acceptable range. Based on this analysis, the optimal asphalt-to-aggregate ratio is determined to be between 6.5% and 8%. Han et al. reported optimal ratios of 7.5–8.6% and 7.4–8.4% using granite and basalt, respectively [35], which are consistent with the findings of this study. The experiment also showed that an asphalt-to-aggregate ratio of 7% avoids asphalt bleeding while adequately coating the aggregates. Therefore, a ratio of 7% is adopted for subsequent experiments.

It is important to note that since this study was conducted in a high-latitude coastal area with lower temperatures and higher humidity, the emulsified asphalt’s breaking time is influenced. Consequently, the mixture design for WER-SBR micro-surfacing has some limitations. Therefore, in practical engineering, it is essential to consider the local climate conditions when selecting an appropriate mixture design for construction.

### 3.2. Wear Resistance Performance

**Figure 12:**
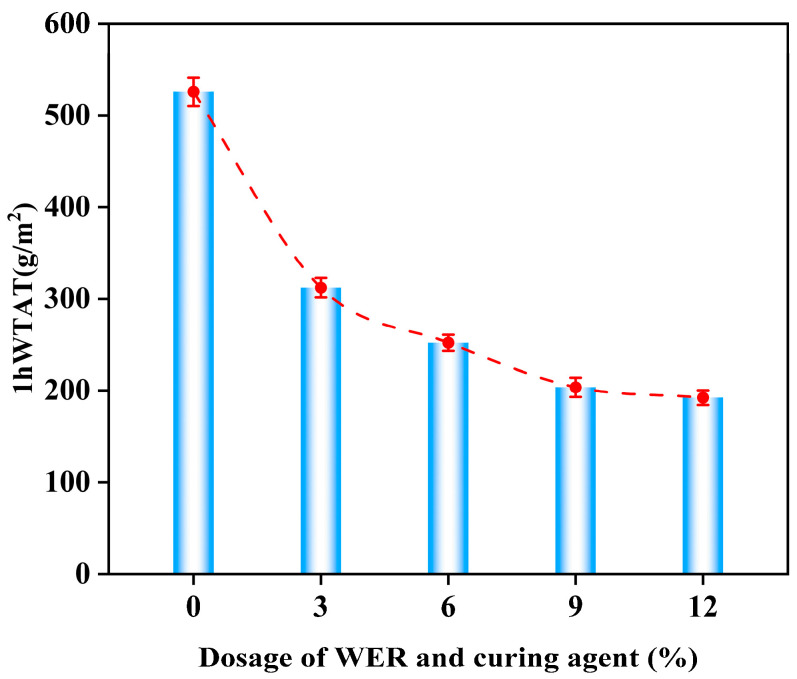
One-hour wet wheel abrasion value curve.

Figure 12 shows the 1 h WTAT of the WER-SBR mixture at different WER contents.

**Figure 13:**
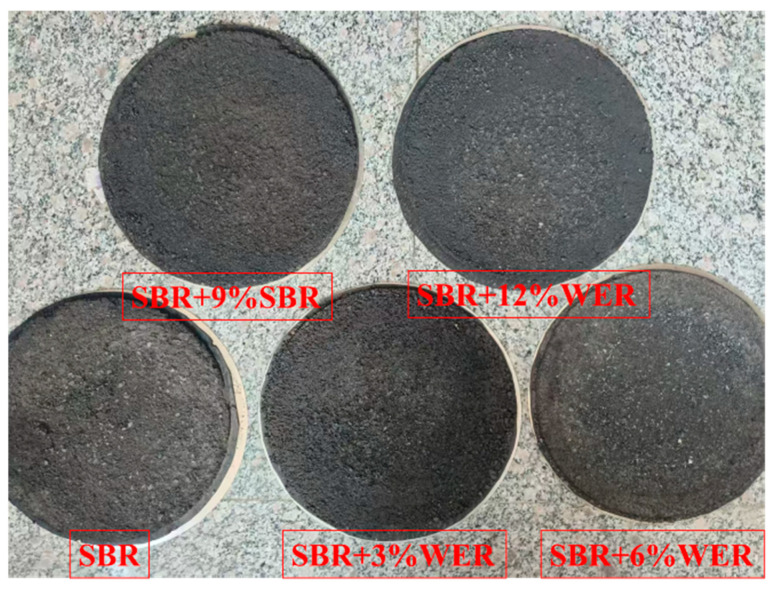
Specimens after abrasion.

The results show that as WER content increases, the 1 h WTAT value of the WER-SBR mixture decreases. At 3%, 6%, 9%, and 12% WER, the WTAT values decreased by 40.6%, 52.0%, 61.3%, and 63.4%, respectively, compared to the SBR mixture. This shows that even a small amount of WER greatly improves the abrasion resistance of the WER-SBR mixture. These results match Huang et al.’s findings [49]. During the experiment, it was observed that the SBR micro-surfacing mixture experienced significant aggregate detachment during abrasion, while the addition of WER resulted in less aggregate loss in the micro-surfacing mixture. The best performance occurred with 6%, 9%, and 12% WER content, as shown in Figure 13. This behavior occurs because as the emulsified asphalt breaks, WER reacts with the curing agent, forming a solid network with the asphalt binder and cement [38,39]. This network structure resists the wear of the abrasion head. When WER content reaches 6%, the 1 h WTAT curve stabilizes, indicating the network structure has reached a stable density, and further increases in WER provide limited strength improvements.

### 3.3. Water Stability

**Figure 14:**
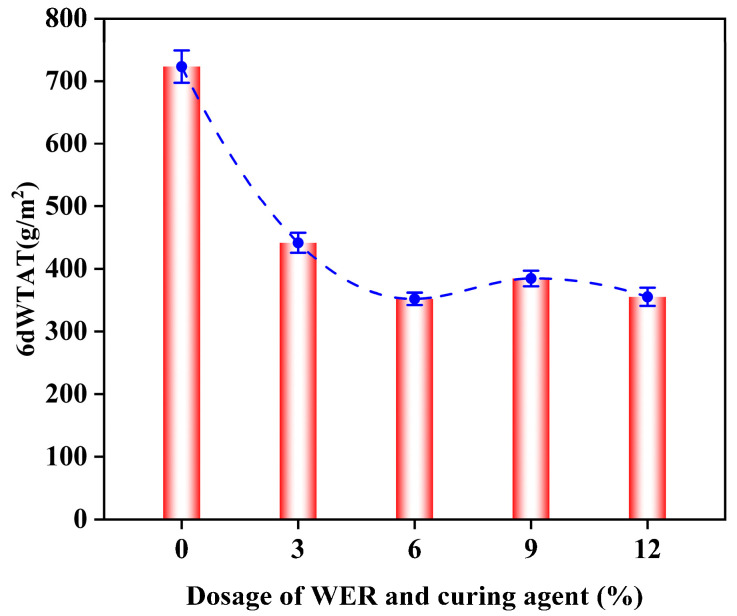
Wet wheel abrasion value curve after 6 days of water immersion.

**Figure 15:**
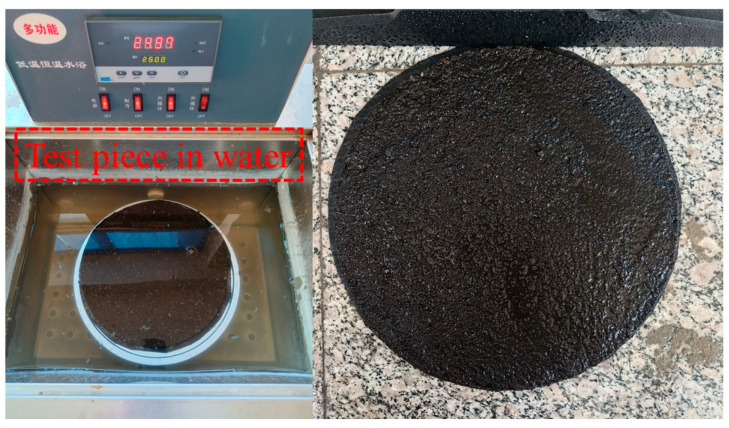
Wet wheel abrasion specimens and post-abrasion specimens under 25 °C constant temperature water bath.

Figure 14 shows the WTAT curves of WER-SBR mixtures after 6 days of water immersion at different WER contents. Figure 15 shows the specimens after 6-day immersion and abrasion.

The results show that as WER content increases, the 6-day WTAT value of the WER-SBR mixture decreases. As WER content increases from 3% to 12%, the 6-day WTAT values decrease by 38.9%, 51.3%, 46.8%, and 50.8%, respectively, compared to the SBR mixture. This shows that adding WER greatly improves the water stability of the mixture [14,49]. This improvement is due to the hydrophobic nature of WER molecules. The polar components of WER adsorb onto the aggregate surface, forming a stable structure that resists water intrusion [26,35,50]. At 6% WER content, the 6-day WTAT value is the lowest. At 9% WER, the 6-day wet track abrasion loss increases slightly but remains low. This suggests that too much WER does not improve the mixture’s resistance to water damage. Therefore, from an economical and practical perspective, it is advisable to limit the maximum WER content to no more than 9%.

### 3.4. Rutting Resistance

**Figure 16:**
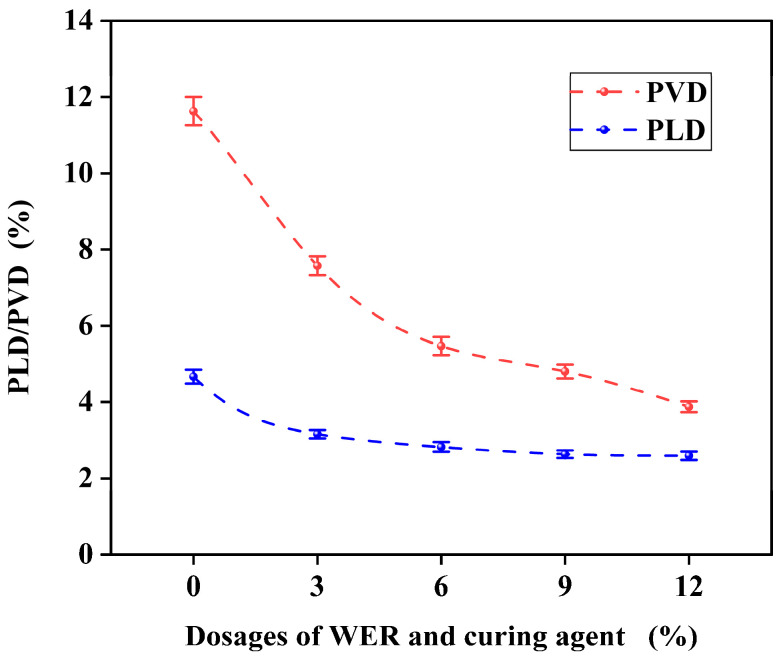
Rutting deformation test results.

Figure 16 shows rutting test results at different WER levels.

**Figure 17:**
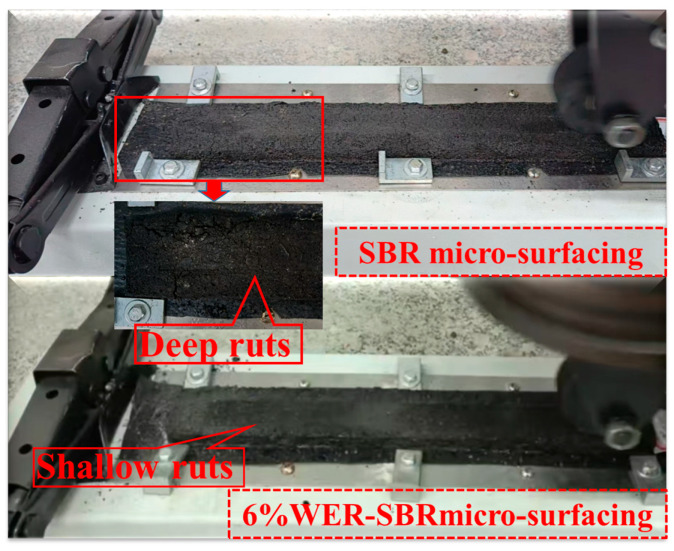
Compacted rutting specimens.

The experimental results indicate that the incorporation of WER into the SBR-based micro-surfacing mixture significantly reduces both the Permanent Lateral Deformation (PLD) and Permanent Vertical Deformation (PVD). As the WER content increases, the decrease in PLD and PVD gradually slows. When the WER dosage increases from 3% to 12%, PLD is reduced by 32.9%, 39.9%, 44.0%, and 45.1%, respectively, demonstrating that the addition of WER substantially enhances the rutting resistance of the micro-surfacing mixture. Similar findings were reported by Liu et al., who achieved comparable results using 11% WER modification [33]. During the rutting tests, it was observed that the SBR-modified micro-surfacing mixture exhibited deeper rut impressions, primarily at both ends of the specimen. This was attributed to the additional impact forces at the specimen edges caused by repeated wheel loading. When the WER content reached 6%, the rut impressions became significantly shallower, as shown in Figure 17. The rutting process can be divided into three stages: compaction, stabilization, and shear deformation. The incorporation of WER helps mitigate the third stage, namely shear deformation [51]. This is because high-speed mixing creates physical cross-links between the emulsified asphalt and WER, while the curing agent forms chemical bonds with WER. These interactions produce a stable, networked resin that prevents asphalt from shearing under high temperatures and heavy loads [36].

### 3.5. Skid Resistance

**Figure 18:**
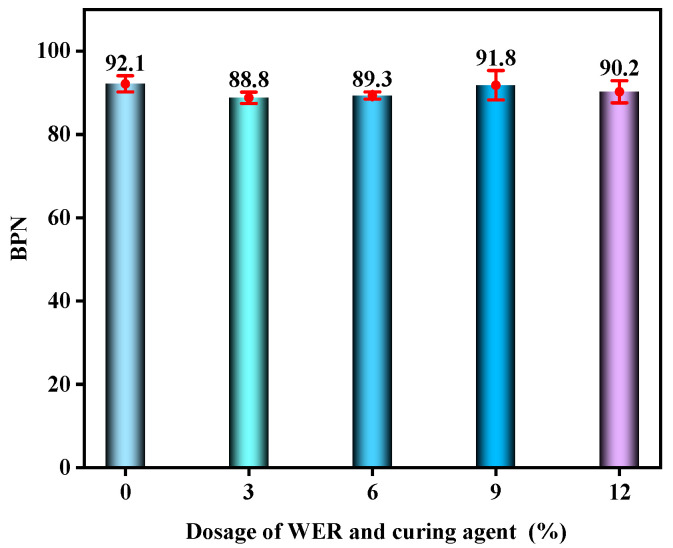
Initial BPN under different WER contents.

**Figure 19:**
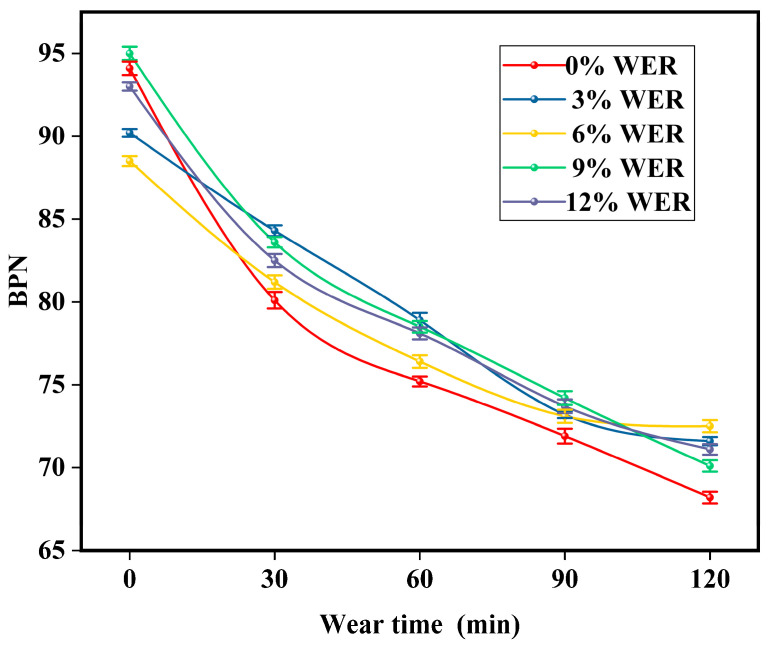
BPN After different durations of abrasion.

The initial BPN of the WER-SBR micro-surfacing mixture under different WER contents is shown in Figure 18. The BPN variation curve after different durations of wet wheel abrasion is shown in Figure 19.

According to the results shown in Figure 17, the BPN values of all specimen groups are significantly higher than the required minimum value of 45, indicating that the WER-SBR micro-surfacing mixtures exhibit good initial skid resistance. However, with increasing WER content, the initial BPN values do not show a clear trend. This suggests that WER has limited influence on improving the initial skid resistance of the mixture during the early stage after paving. After a period of abrasion, the skid resistance of the mixtures begins to decline. The BPN values drop sharply within the first 30 min of abrasion, then gradually stabilize. This behavior is due to the surface of the WER-SBR micro-surfacing mixture being quickly polished under abrasive action, resulting in reduced surface texture and skid resistance. As abrasion continues, the degree of surface wear tends to stabilize, causing the BPN reduction rate to slow down [35,52]. As shown in Figure 18, the BPN curves of specimens with 0% and 9% WER rapidly decrease, while those with 3% and 6% WER show slower rates of decline. This indicates that the addition of WER improves the skid resistance of the WER-SBR micro-surfacing mixture after abrasion. However, the WER content should not be too high. Huang et al. suggested that the content of hard WER should be kept below 10% [49]. This finding is consistent with the results of this study.

### 3.6. Low-Temperature Crack Resistance

**Figure 20:**
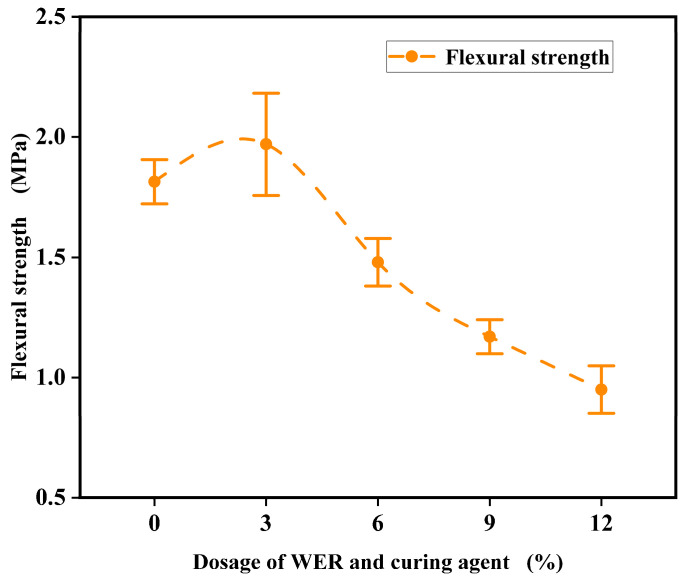
Semi-circular bending test results.

The semi-circular bending test results of the WER-SBR micro-surfacing mixture under different WER contents are shown in Figure 20.

**Figure 21:**
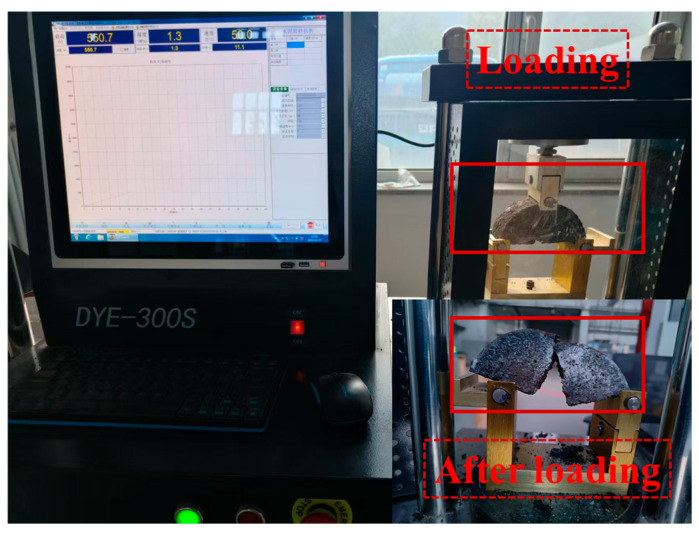
Specimen loading process.

The test results indicate that as the WER content increases, the bending strength of the WER-SBR micro-surfacing semi-circular specimens generally decreases. A slight increase is observed when the WER content reaches 3%, but at 6% WER, the bending strength gradually decreases, with a reduction of 0.335 MPa compared to 0% WER. The decrease is relatively small. During testing, it was observed that specimens with 0% and 3% WER exhibited slow crack propagation upward from the pre-cut notch, which aligns with the findings of Yao et al. [53]. In contrast, specimens with WER content above 6% showed brittle fracture behavior, as illustrated in Figure 21. The addition of WER increases the low-temperature stiffness of the asphalt. The high stiffness of WER curing products severely limits the mixture’s deformation at low temperatures. As a result, stress relaxation is difficult, leading to stress accumulation and increased risk of low-temperature cracking in the pavement [31]. It is important to note that when used as a pavement-wearing course, the thickness of the micro-surfacing mixture typically does not exceed 20 mm. However, the SCB specimens are thicker, which may cause an inconsistency in stress distribution compared to the actual response of thin-layer structures. Therefore, they may not accurately reflect the true cracking resistance of the thin-layer material, presenting certain limitations.

### 3.7. Durability Performance

#### 3.7.1. Freeze–Thaw Test Results

**Figure 22:**
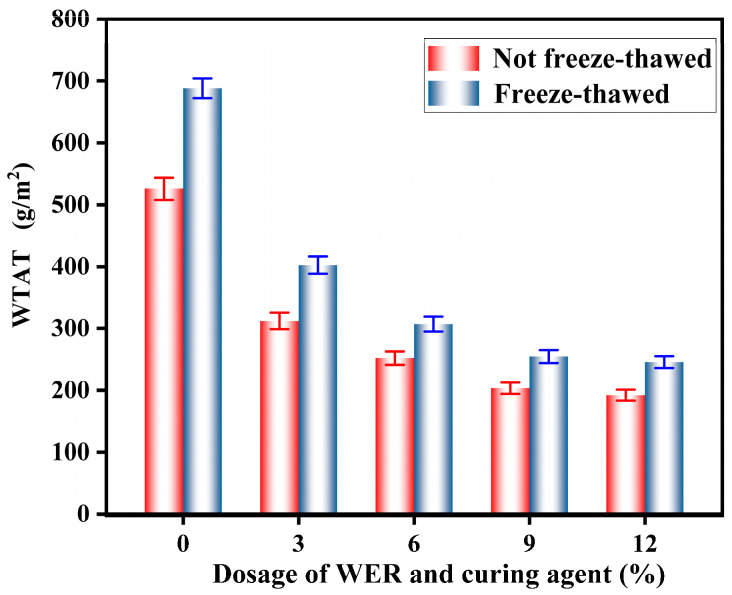
Freeze–thaw cycle test results.

**Figure 23:**
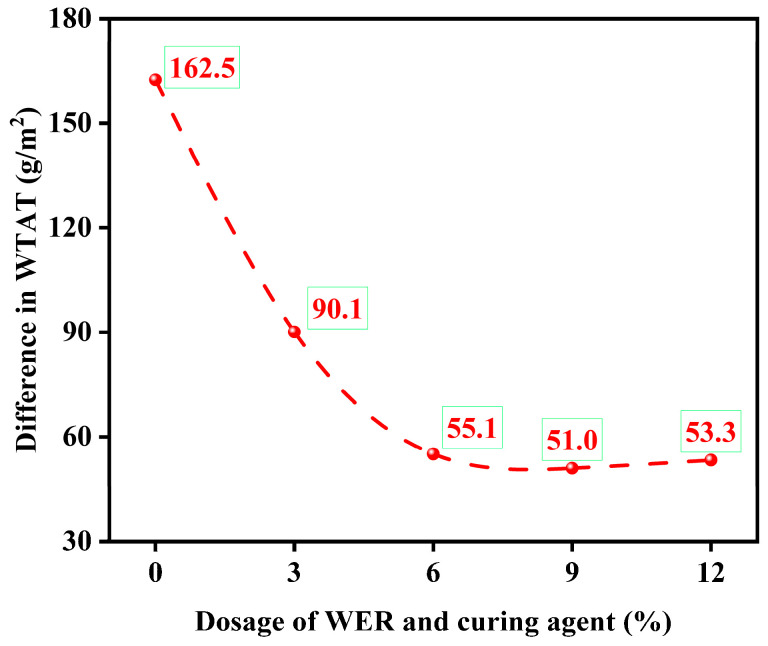
Freeze–thaw loss difference curve.

The WTAT results of the WER-SBR micro-surfacing mixture after the freeze–thaw test are shown in Figure 22. The curve of the wet wheel abrasion loss difference after freeze–thaw cycles are plotted in Figure 23.

The test results show that as the amount of WER increases, the wet wheel abrasion loss of the WER-SBR micro-surfacing mixture decreases both in freeze–thaw and non-freeze–thaw cycles. Additionally, the difference in wet wheel abrasion loss after freeze–thaw cycles (ΔWTAT) gradually decreases. When the WER content reaches 6%, ΔWTAT stabilizes around 50 g/m^2^. The addition of WER enhances the freeze–thaw resistance of the micro-surfacing mixture. However, when the WER content reaches 6%, further improvements in frost resistance become limited. Liu et al. observed similar trends in freeze–thaw tests of fog seal and micro-surfacing layers [34,54]. This can be attributed to the dense, cross-linked spatial network structure formed by WER after curing, which is free from hydrophilic impurities. This structure helps retain asphalt molecules, preventing them from being displaced by water and enhances the adhesion of the asphalt binder. As a result, stronger bonding is formed between the asphalt and aggregate particles [26,38].

#### 3.7.2. Dry–Wet Test Results

**Figure 24:**
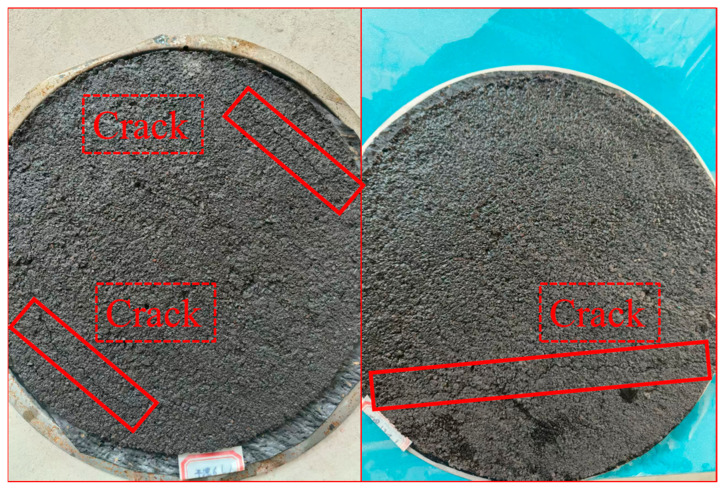
Crack conditions of specimens after dry–wet cycles.

**Figure 25:**
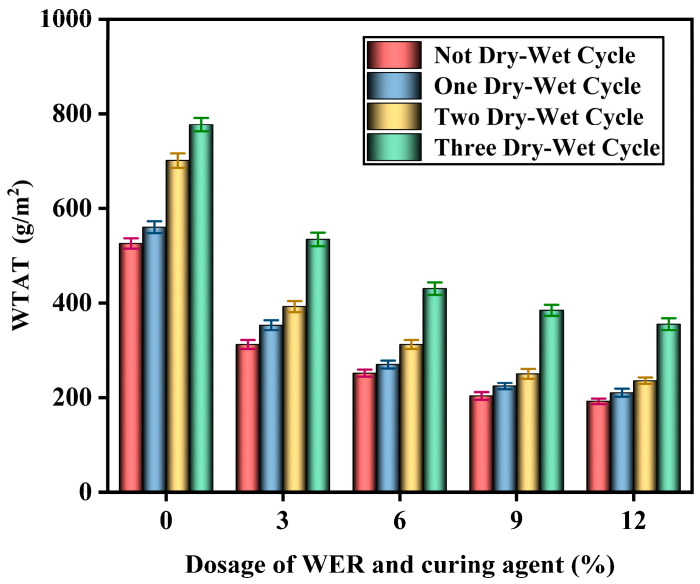
Dry–wet cycle test results.

The surface crack conditions of the specimens after a certain number of dry–wet cycles are shown in Figure 24, and the WTAT test results of the WER-SBR micro-surfacing mixture after different dry–wet cycles are shown in Figure 25.

The dry–wet cycling test included six stages: water injection, soaking, drainage, air drying, oven drying, and cooling. Each cycle lasted 24 h. After the first cycle, slight cracks appeared at the edges of all specimens. As the number of cycles increased, the cracks gradually spread toward the center. After three cycles, visible cracking was observed. This damage was caused by the high drying temperature, which rapidly removed pore water from the mixture, weakening the adhesion between asphalt and aggregates. Test results indicate that as the WER content increased, the WTAT values of the WER-SBR micro-surfacing mixtures decreased. After dry–wet cycling, WTAT values increased to varying extents. The third cycle showed a significant increase in WTAT, with the SBR micro-surfacing mixture exhibiting the largest rise—an increase of 251.3 g/m^2^ compared to before cycling. In contrast, mixtures with 6% and 12% WER showed smaller increases of 176.0 g/m^2^ and 163.3 g/m^2^, respectively. These findings suggest that WER molecules can effectively infiltrate and densify the mixture’s microscopic pore structure, forming an elastic interfacial transition zone under alternating dry–wet conditions. This structure significantly reduces the capillary water infiltration effect [50,55].

#### 3.7.3. Salt Spray Test Results

**Figure 26:**
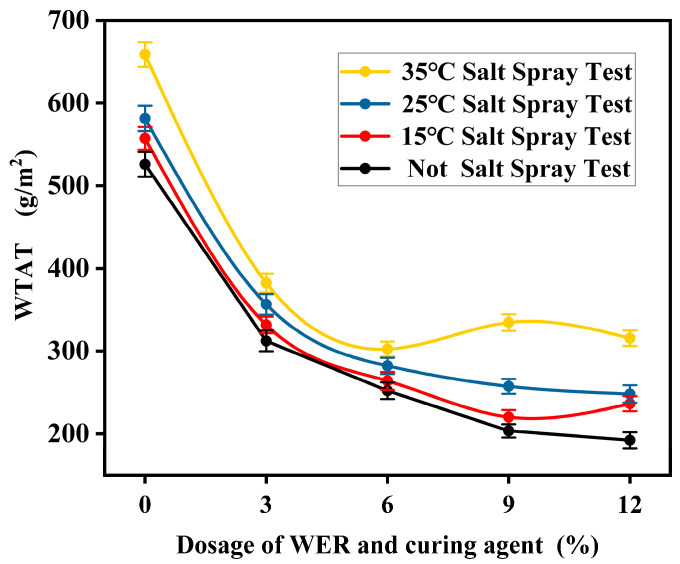
Salt fog test results.

The WTAT results of the WER-SBR micro-surfacing mixture after undergoing salt spray tests at different temperatures are shown in Figure 26.

The test results indicate that with the increase in WER content, the WTAT values of the specimens after salt spray exposure generally show a decreasing trend. When the WER content is between 3% and 6%, the difference in WTAT before and after salt spray exposure is minimal. This indicates that the spatial network formed by WER and SBR effectively blocks chloride ion intrusion, enhancing the mixture’s resistance to salt spray. Thus, the damaging effects of the salt spray environment are significantly reduced. When the WER content reaches 6%, the difference in WTAT at 35 °C before and after salt spray exposure reaches its lowest point. When WER content exceeds 6%, its resistance to chloride ions begins to weaken. This is because the high dosage of WER and curing agent causes the modified asphalt to cure rapidly during mixing [44], preventing full coating of the aggregates. As a result, internal voids increase, reducing the mixture’s resistance to salt spray. The test results also show that wet track abrasion values increased with rising salt spray curing temperatures. This indicates that high-temperature salt spray allows chloride ions to penetrate the mixture more easily, reducing its abrasion resistance.

### 3.8. Microscopic Morphology

**Figure 27:**
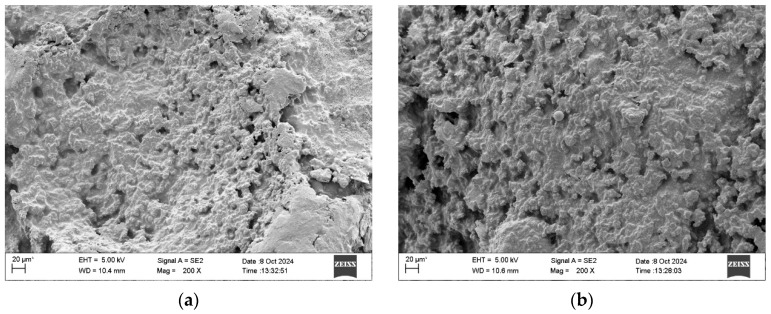

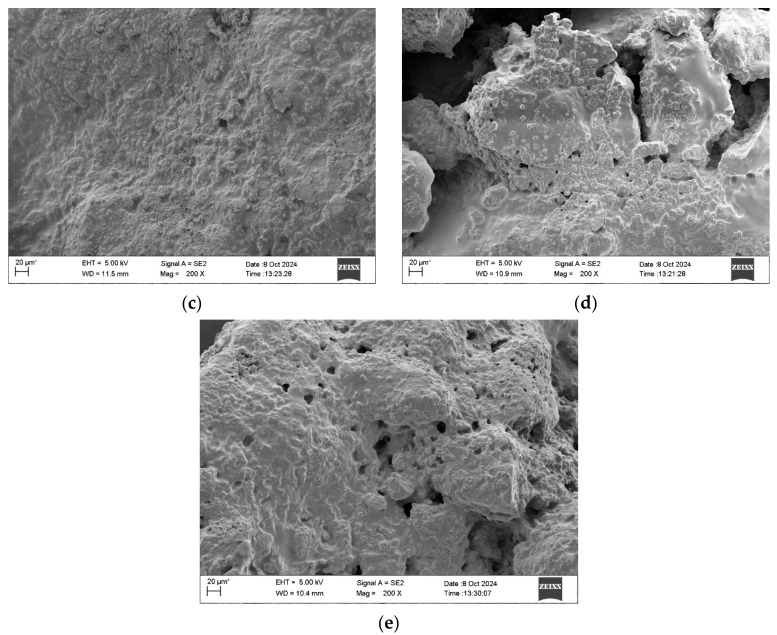
Surface morphology of WER-SBR micro-surfacing mixture with different WER contents: (**a**) 0%; (**b**) 3%; (**c**) 6%; (**d**) 9%; (**e**) 12%.

The microscopic morphology of the surface of WER-SBR micro-surfacing mixture specimens with different WER contents is shown in Figure 27.

As shown in Figure 27a,b, at 0% and 3% WER, loose aggregates remain on the specimen surface, and voids between particles are not fully filled with asphalt slurry. The low WER content results in weak asphalt–aggregate adhesion, preventing the formation of a continuous asphalt film. As a result, the mixture cannot resist water infiltration or chloride attack, leading to poor overall performance. At a WER content of 6%, as shown in Figure 27c, a complete and relatively uniform asphalt film forms on the specimen surface. The voids between aggregates and asphalt are adequately filled, and the internal cohesion is enhanced, thereby improving the interfacial transition zone between asphalt and aggregates. This results in excellent pavement performance and durability. These findings are consistent with those reported by other researchers regarding the compatibility of WER with asphalt emulsions [22,25,38,44,49]. When the WER content increases to 9% and 12%, as illustrated in Figure 27d,e, the specimen surface showed visible honeycombing and cracking, with a thick and uneven asphalt film. This occurred because excessive WER caused rapid curing, preventing the asphalt from properly coating the aggregates. Much of it solidified into clumps, reducing low-temperature crack resistance and limiting improvements in water stability and durability.

## 4. Conclusions

This study investigates a micro-surfacing mixture designed for the winter climate in high-latitude coastal areas. High-quality WER was added to modify the SBR micro-surfacing mixture. The performance of the WER-SBR micro-surfacing mixture was tested for abrasion, rutting, skid resistance, bending, and durability. Scanning electron microscopy revealed the strengthening mechanism of WER in the mixture, correlating with macroscopic test results. Finally, the optimal WER content was determined. Based on the experimental results, the following conclusions can be drawn:

- The 1 h WTAT of WER-SBR mixtures decreases as the binder-to-aggregate ratio increases while LWT rises. The comprehensive binder-to-aggregate ratio range for WER-SBR mixtures is determined to be 6.5–8%. Based on the sample morphology, the binder-to-aggregate ratio selected in this study was 7%.
- Increasing WER content improves wear resistance, water stability, and rutting resistance. Improvements become less significant when WER content exceeds 6%. In terms of skid resistance and cracking resistance, WER-SBR mixtures with 3% and 6% WER exhibit slower BPN decay rates, and the low-temperature bending stress remains at a higher level.
- After freeze–thaw, wet–dry, and salt fog tests, the WTAT of WER-SBR mixtures increases. After the addition of WER, the WTAT of the mixtures starts to decrease. When the WER content reaches 6%, the difference in WTAT before and after the tests is minimized.
- SEM tests show that at 0% and 3% WER, the surfaces have loose aggregates. At 6% WER, a complete asphalt film forms on the surface. At 9% and 12% WER, the specimens exhibit cracking and void formation.
- The addition of 6% WER to SBR mixtures improves resistance to wheel wear, moisture penetration, rutting, and damage from extreme conditions like freeze–thaw, wet–dry cycles, and salt fog. The mixture demonstrates optimal road performance and durability. Therefore, the recommended WER content is 6%.

This work determines the optimal WER content in SBR mixtures and explains the microscopic mechanisms. The findings contribute to the application of micro-surfacing technology in cold regions and help extend the service life of mixtures.

While this study has achieved results, some issues were not explored due to time and resource limitations. Future research could explore the following areas. In terms of optimizing micro-surfacing materials, waste materials such as steel slag [56], iron tailings, and phosphogypsum could be introduced as fillers to reduce material costs and provide high environmental benefits. In terms of mechanism analysis, molecular dynamics simulation methods could be used to analyze the curing and modification mechanisms of WER at the atomic scale, providing theoretical support for the application of polymers in material engineering.

## Figures and Tables

**Table 1:** Emulsified asphalt technical specifications.

Technical Parameters	Units	Values	Standard
Penetration (25 °C)	0.1 mm	90.5	45–150
Evaporation Residue Content	%	57.5	≥55
Ductility (15 °C)	cm	116	≥40
Standard Viscosity	S	15.6	10–60
Solubility	%	97.7	≥97.5
5-Day Storage Stability	%	0.8	≤5
Sieve Residue	%	0.02	≤0.1

**Table 2:** The technical specifications of waterborne epoxy resin and curing agent.

Modifier	Parameters	Units	Values
WER	Appearance	-	Milky white
	epoxy equivalent	G/EQ	400–800
	rotational viscosity	Pa·s	<1000
	solid content	%	47–53
	pH value	-	4.3
	specific gravity	-	1.01–1.08
Curing agent	Appearance	-	Pale yellow
	rotational viscosity	Pa·s	>2000
	solid content	%	42–46
	pH value	-	10.2
	specific gravity	-	1.00–1.08

**Table 3:** Aggregate technical specifications.

Modifier	Parameters	Units	Values	Standard
Coarse aggregate	Crushing value	%	10.8	≤26
	Los Angeles abrasion loss	%	9.2	≤25
	Polish value	BPN	50	≥42
	Toughness	%	10.8	≤12
	Flakiness content	%	12.1	≤15
Fine aggregate	Toughness	%	10.8	≤12

**Table 4:** Filler technical specifications.

Modifier	Parameters	Units	Values	Standard
Limestone powder	Moisture content	%	0.3	≤1
	Apparent relative density	-	2.95	≥2.50
	Hydrophilicity index	-	0.4	<1
	Plasticity index	%	3	<4
Cement	Specific surface area	m^2^/kg	341	≥300
	Density	kg/m^3^	3065	-
	Initial setting time	min	176	≥45
	Final setting time	min	284	≤600
	3-Day compressive strength	MPa	28.5	≥17
	3-Day flexural strength	MPa	4.8	≥3.5

**Table 5:** Material proportions of experimental groups with different WER contents.

Experimental Group	Aggregate (g)	Mineral Filler (g)	Cement (g)	Water (g)	Modified Asphalt		
					BC-1 Emulsified Asphalt (g)	WER System (g)	SBR Latex (g)
0%WER + 3%SBR	100	4	2	7	6.79	0	0.21
3%WER + 3%SBR	100	4	2	7	6.58	0.21	0.21
6%WER + 3%SBR	100	4	2	7	6.37	0.42	0.21
9%WER + 3%SBR	100	4	2	7	6.16	0.63	0.21
12%WER + 3%SBR	100	4	2	7	5.95	0.84	0.21

## Data Availability

The original contributions presented in the study are included in the article; further inquiries can be directed to the corresponding author.
